# Supplementary material for: Glycan chip based on structure-switchable DNA linker for on-chip biosynthesis of cancer-associated complex glycans
Source: Nat Commun. 2021 Mar 2;12:1395. doi: 10.1038/s41467-021-21538-0 (PMC7925590; doi:10.1038/s41467-021-21538-0)
Supplement: Supplementary file 1 — Supplementary Information [file 41467_2021_21538_MOESM1_ESM.pdf]

SUPPORTING INFORMATION

Glycan chip based on structure-switchable DNA linker for on-chip biosynthesis of cancer-associated complex glycans

Hye Ryoung Heo[1,2], Kye Il Joo[2], Jeong Hyun Seo[3],

Chang Sup Kim[4*] & Hyung Joon Cha[1,2*]

[1]*School of Interdisciplinary Bioscience and Bioengineering, Pohang University of Science and Technology, Pohang 37673, Korea*

[2]*Department of Chemical Engineering, Pohang University of Science and Technology, Pohang 37673, Korea*

[3]*School of Chemical Engineering, Yeungnam University, Gyeongsan 38541, Korea*

[4]*School of Chemistry and Biochemistry, Yeungnam University, Gyeongsan 38541, Korea*

*Corresponding author: hjcha@postech.ac.kr (H.J. Cha); cskim1409@ynu.ac.kr (C.S. Kim)

Supplementary Table 1. Oligonucleotides used in this study and their sequences

DNA	Sequence[a]
i-motif DNA	HS-5'- TTTTTTTTTTCCCTAACCCTAACCCTAACCC-3'
Complementary ssDNA	3'-G**<u>T</u>**GATTGGGATT**<u>T</u>**GGATTG**<u>T</u>**G-5'-SH

[a]Bold underlines present three mismatch bases with i-motif DNA.

Supplementary Table 2. Quantitative analysis of relative conversion efficiency for on-surface biosynthesized complex glycans in Supplementary Figs. 9-11

Reaction time (h)	Relative conversion efficiency (%)		
	**GM3 trisaccharide**	**GM2 tetrasaccharide**	**GM1 pentasaccharide**
12	-	-	-
24	91.91	-	-
48	93.77	-	-
72	-	88.11	0.84
120	-	90.64	1.58
168	-	92.32	-

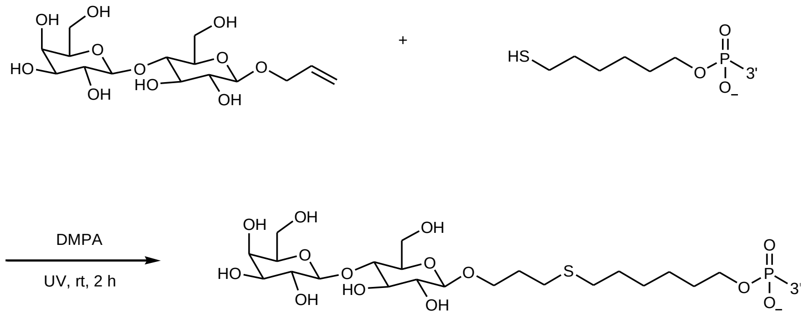

Supplementary Figure 1. Synthesis of lactose-oligonucleotide conjugates.

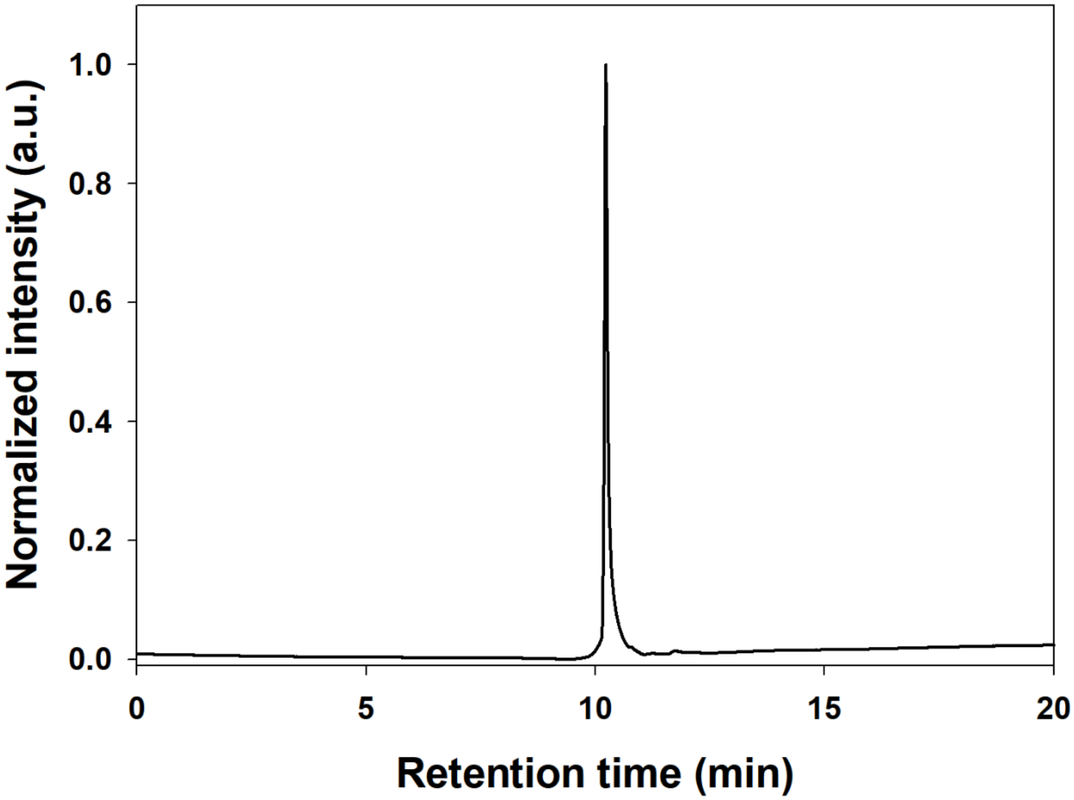

Supplementary Figure 2. Purification of lactose-oligonucleotide conjugates using HPLC. Source data are provided as a Source Data file.

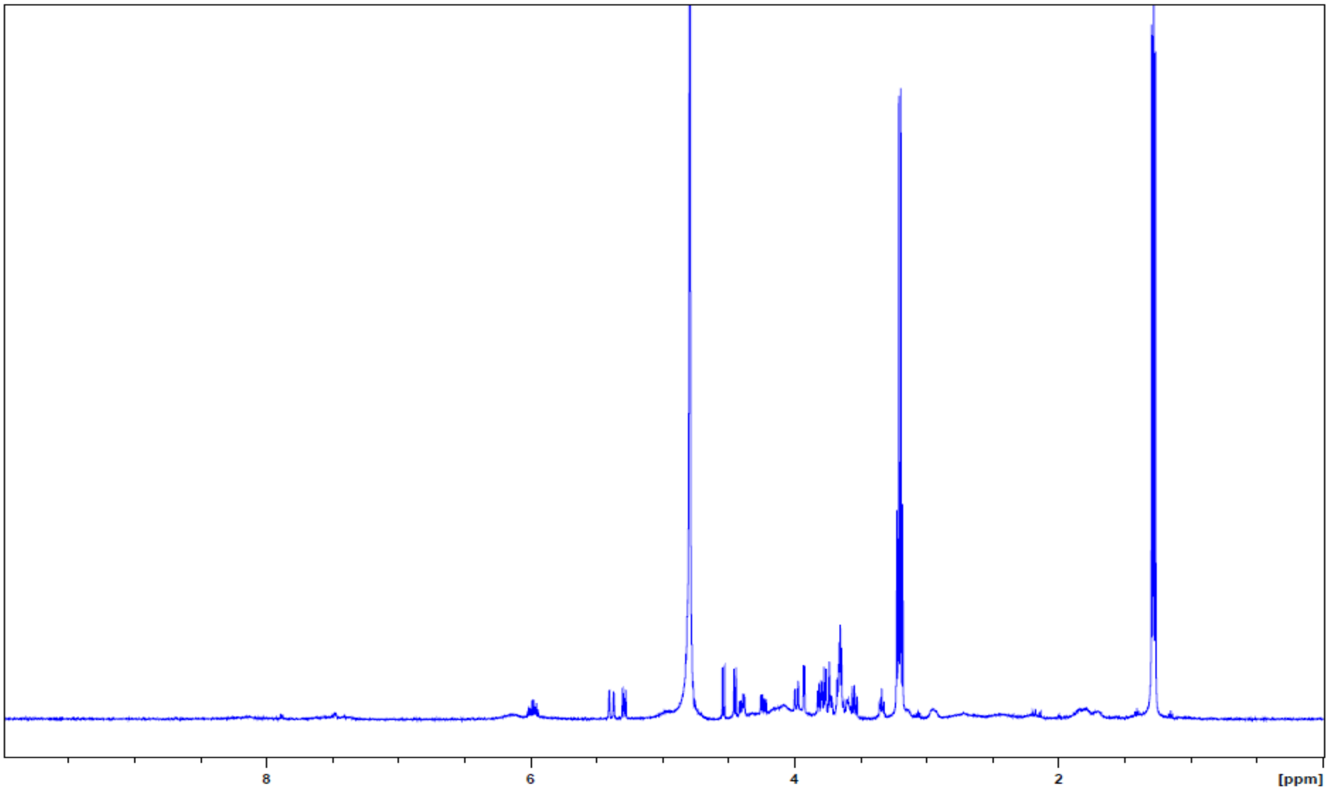

Supplementary Figure 3. ^{1}H NMR spectrum of lactose-oligonucleotide conjugates. ^{1}H NMR (500 MHz, D_2O): δ 8.26-7.34 (m, oligonucleotide-H), 6.19-6.06 (m, oligonucleotide-H), 6.03-5.95 (m, 2H), 5.41-5.38 (dd, J = 17.2, 1.3 Hz, 1H), 5.31-5.29 (d, J = 10.7 Hz, 1H), 4.55 (d, J = 8 Hz, 1H), 4.46 (d, J = 7.8 Hz, 1H), 4.43-4.34 (m, 1H), 4.26-4.22 (m, 1H), 4.01-3.98 (dd, J = 12.3, 2 Hz, 1H), 3.94 (d, J = 3.3 Hz, 1H), 3.83-3.58 (m, 8H), 3.57-3.53 (t, J = 8.9 Hz, 1H), 3.36-3.33 (t, J = 8.6 Hz, 1H), 3.23-3.19 (q, oligonucleotide-H, J = 7.3 Hz), 1.30-1.27 (t, oligonucleotide-H, J = 7.4 Hz).

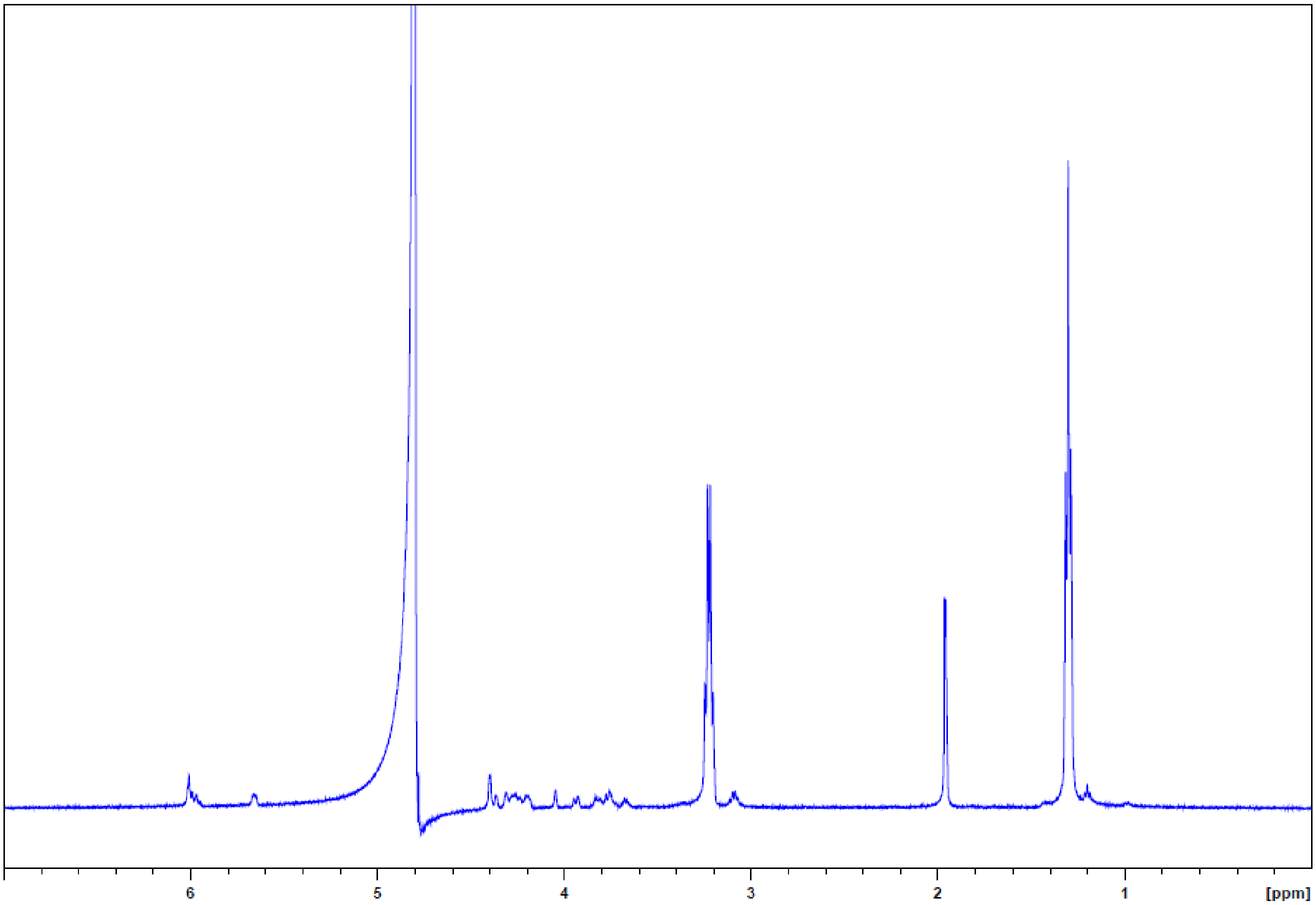

Supplementary Figure 4. ^{1}H NMR spectrum of Gb3-oligonucleotide conjugates. ^{1}H NMR (500 MHz, D_2O): δ 6.01 (m, 1H), 6.00-5.99 (dd, J = 20.4, 8.5 Hz, 3H), 5.67-5.65 (m, 2H), 4.40-4.39 (d, J = 2.9 Hz, 2H), 4.36-4.35 (d, J = 2.5 Hz, 1H), 4.31-4.18 (m, 6H), 4.05 (s, 1H), 3.95-3.92 (dd, J = 10.1, 2.5 Hz, 2H), 3.83-3.74 (m, 5 H), 3.68-3.67 (d, J = 6.5 Hz, 2H), 3.37-3.36 (m, 1H), 3.25-3.20 (q, oligonucleotide-H, J = 7.2 Hz), 1.32-1.29 (t, oligonucleotide-H, J = 7.2 Hz).

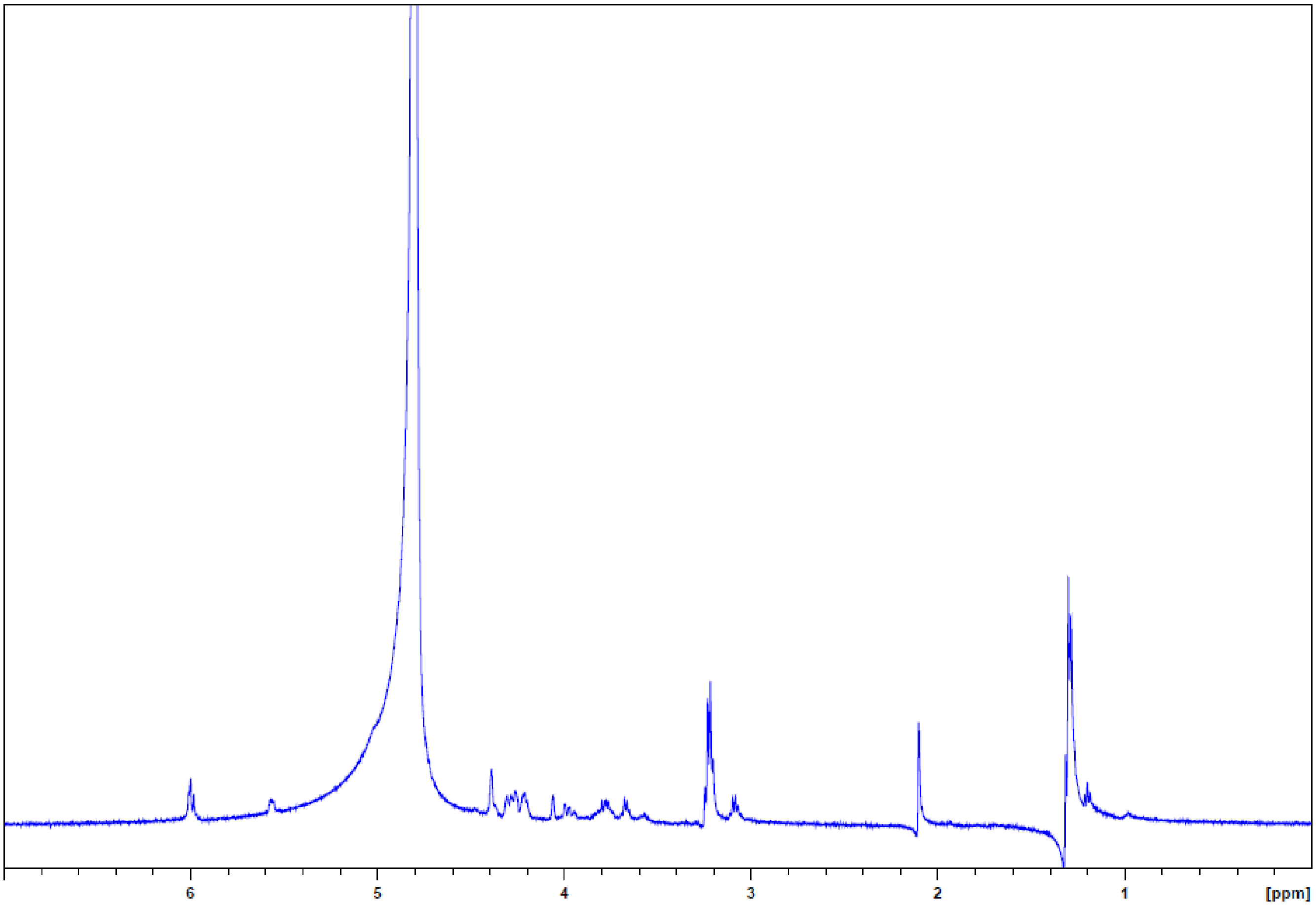

Supplementary Figure 5. ^{1}H NMR spectrum of Gb4-oligonucleotide conjugates. ^{1}H NMR (500 MHz, D_2O): δ 6.01 (m, 1H), 6.00-5.98 (d, J = 8.6 Hz, 3H), 5.58-5.56 (m, 2H), 4.48-4.47 (d, J = 6.5 Hz, 1H), 4.40-4.37 (m, 4H), 4.31-4.26 (m, 5H), 4.22-4.20 (m, 3H), 4.06-4.05 (d, J = 2.5 Hz, 2H), 4.00-3.95 (m, 3H), 3.88-3.72 (m, 5H), 3.69-3.65 (m, 3H), 3.59-3.55 (m, 1H), 3.29-3.28 (m, 1H), 2.10 (s, 3H), 3.25-3.20(q, oligonucleotide-H, J = 7.2 Hz), 1.32-1.29 (t, oligonucleotide-H, J = 7.0 Hz).

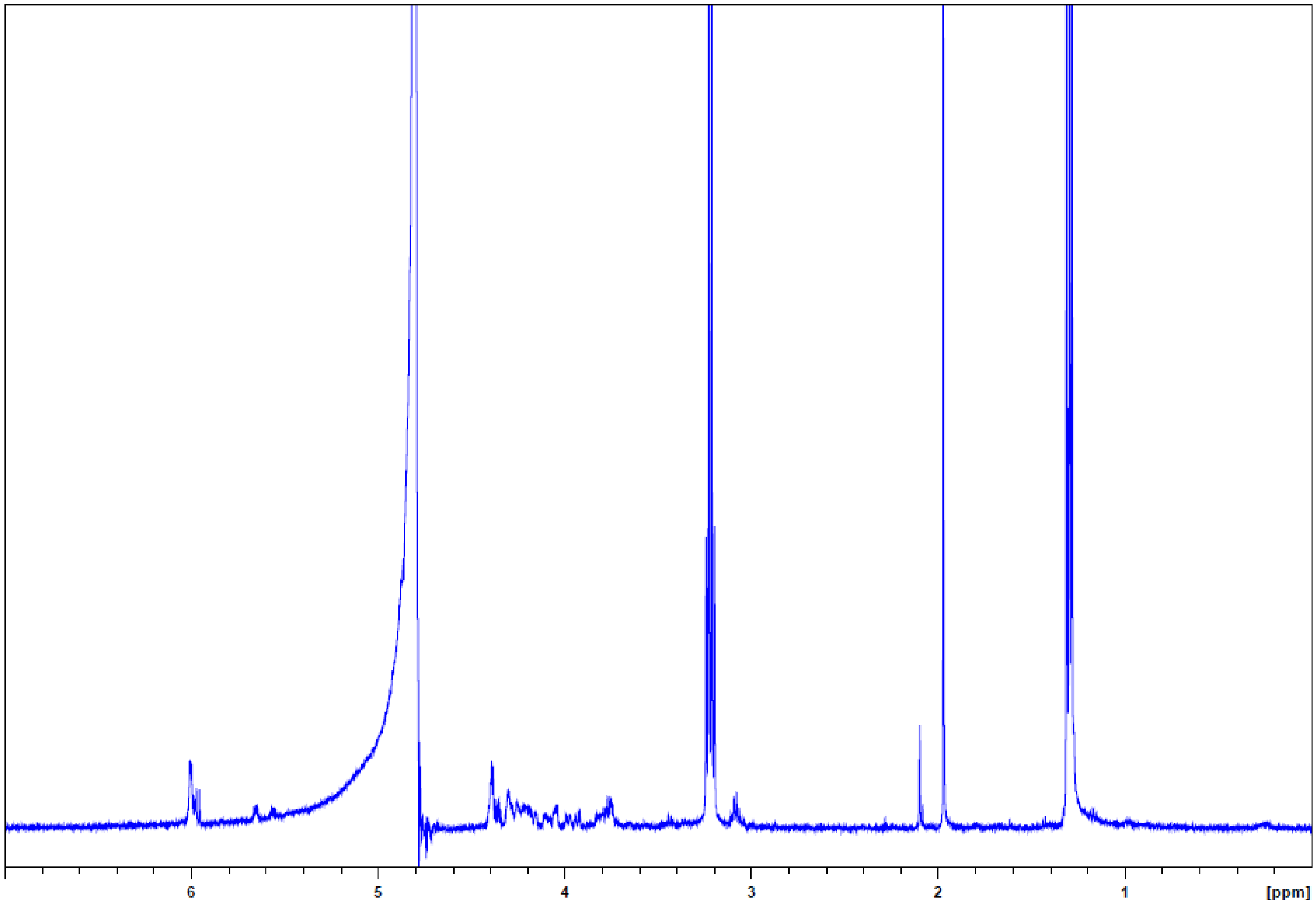

Supplementary Figure 6. ^{1}H NMR spectrum of Gb5-oligonucleotide conjugates. ^{1}H NMR (500 MHz, D_2O): δ 6.00 (m, 5H), 5.67-5.64 (dd, J = 12.6, 3.8 Hz, 2H), 5.57-5.55 (dd, J = 7.1, 3.4 Hz, 2H), 4.40-4.38 (m, 6H), 4.36-4.34 (m, 2H), 4.30-4.29 (m, 5H), 4.26-4.25 (m, 2H), 4.23-4.18 (m, 4H), 4.15 (m, 1H), 4.11-4.10 (m, 1H), 4.06-4.04 (m, 2H), 4.00-3.97 (dd, J = 10.7, 3.5 Hz, 1H), 3.95-3.92 (dd, J = 10.2, 3.4 Hz, 1H), 3.83-3.74 (m, 6H), 3.45-3.43 (d, J = 7.9 Hz, 1H), 2.10 (s, 3H), 3.24-3.20(q, oligonucleotide-H, J = 7.4 Hz), 1.31-1.28 (t, oligonucleotide-H, J = 7.3 Hz).

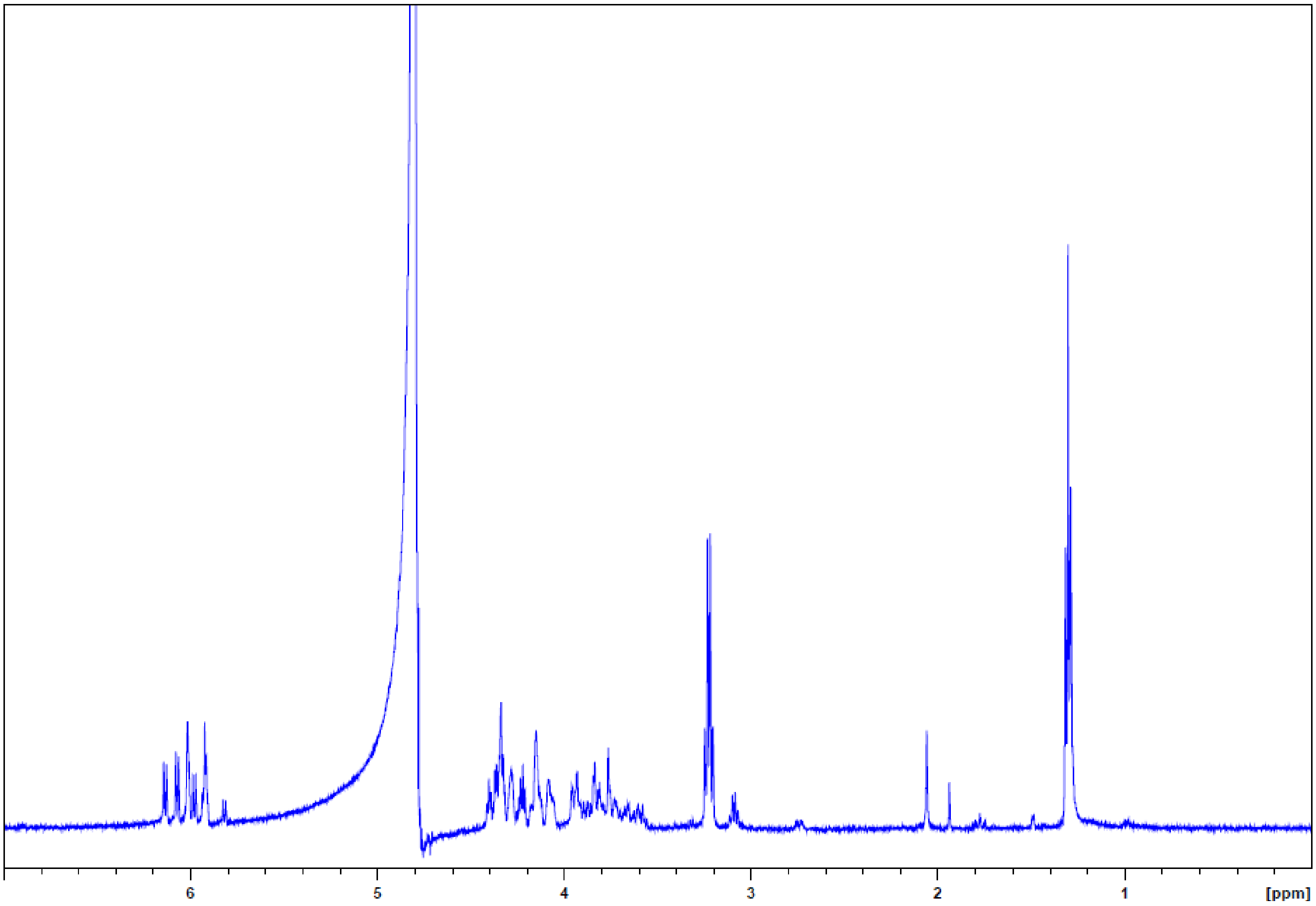

Supplementary Figure 7. ^{1}H NMR spectrum of SSEA-4-oligonucleotide conjugates. ^{1}H NMR (500 MHz, D_2O): δ 6.00 (s, 1H), 5.98 (s, 1H), 5.96-5.94 (d, J = 8 Hz, 2H), 5.81-5.80 (d, J = 7.7 Hz, 2H), 4.33-4.32 (m, 10H), 4.29-4.28 (m, 4H), 4.22-4.21 (m, 4H), 4.20 (m, 2H), 4.15-4.01 (m, 4H), 3.95-3.94 (d, J = 2.7 Hz, 2H), 3.93-3.92 (d, J = 2.8 Hz, 2H), 3.83-3.79 (m, 7H), 3.75-3.74 (m, 5H), 2.74-2.71 (dd, J = 14.6, 4.7 Hz, 1H), 2.23 (s, 3H), 2.04 (s, 3H), 1.78-1.74 (t, J = 12 Hz, 1H), 3.23-3.19(q, oligonucleotide-H, J = 7.3 Hz), 1.30-1.27 (t, oligonucleotide-H, J = 7.3 Hz).

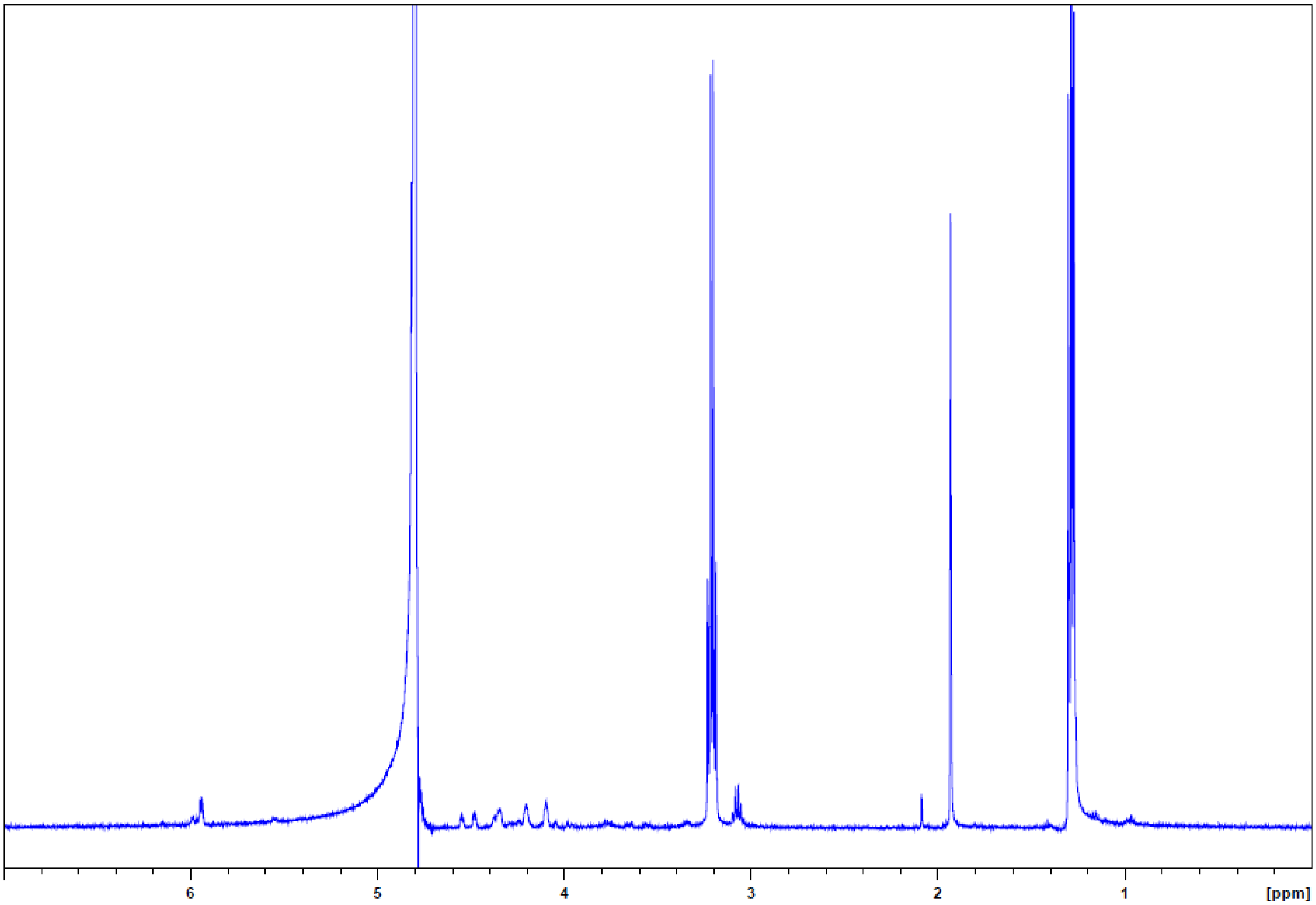

Supplementary Figure 8. ^{1}H NMR spectrum of Globo H-oligonucleotide conjugates. ^{1}H NMR (500 MHz, D_2O): δ 6.01 (m, 1H), 5.95-5.94 (d, J = 2.15 Hz, 3H), 5.94-5.93 (d, J = 2.4 Hz, 3H), 5.55-5.54 (m, 1H), 4.56-4.54 (m, 3H), 4.49-4.47 (m, 3H), 4.38-4.37 (m, 2H), 4.36-4.34 (m, 6H), 4.30-4.29 (m, 1H), 4.27-4.24 (m, 2H), 4.21-4.20 (m, 7H), 4.10-4.09 (m, 7H), 4.04 (d, J = 2.9 Hz, 1H), 3.98-3.96 (m, 1H), 3.78-3.76 (d, J = 7.4 Hz, 2H), 3.67-3.63 (m, 1H), 3.58-3.56 (m, 1H), 3.36-3.33 (m, 1H), 2.09 (s, 3H), 1.20-1.19 (d, J = 7.1 Hz, 3H), 3.23-3.19(q, oligonucleotide-H, J = 7.3 Hz), 1.30-1.27 (t, oligonucleotide-H, J = 7.3 Hz).

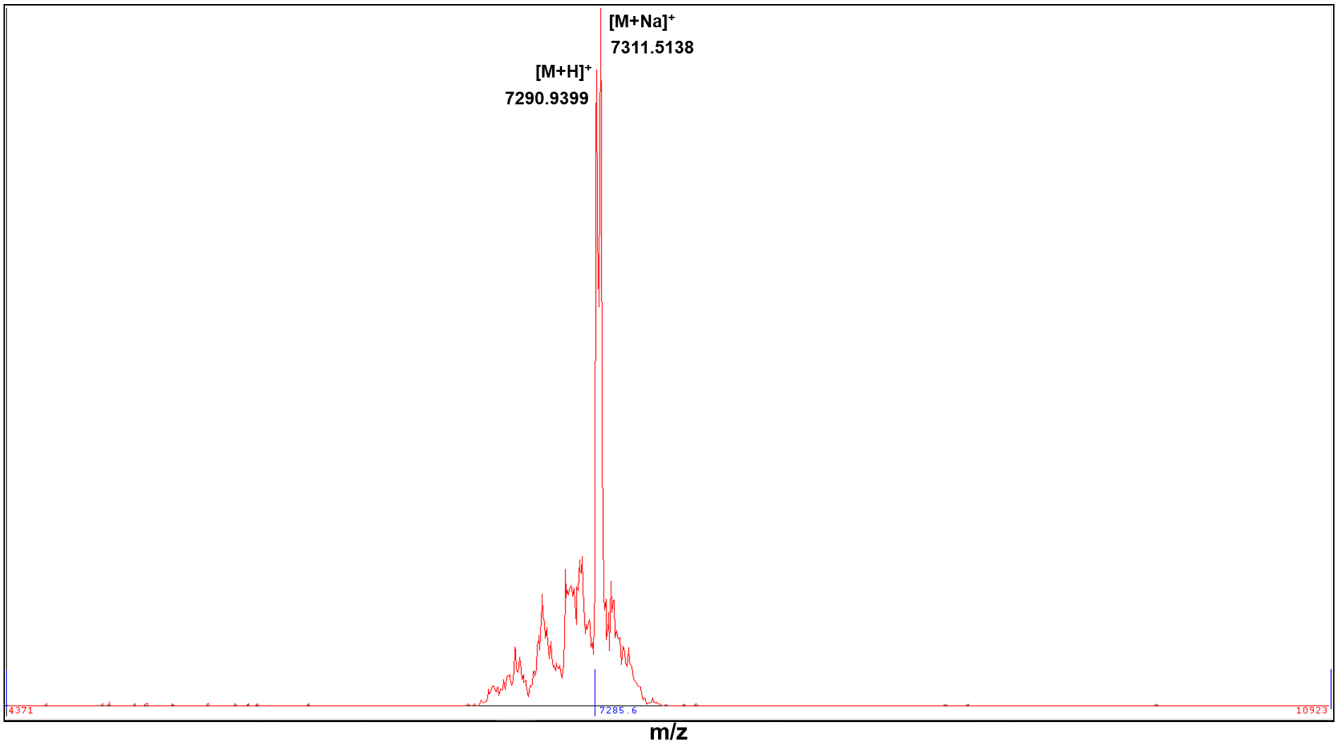

Supplementary Figure 9. **MALDI-TOF MS analysis of lactose-oligonucleotide conjugates.** MALDI-TOF MS (m/z): $[M+H]^+$ calcd. for lactose-oligonucleotide conjugate, 7289.47; found, 7290.94; $[M+Na]^+$ calcd. 7312.46; found, 7311.51.

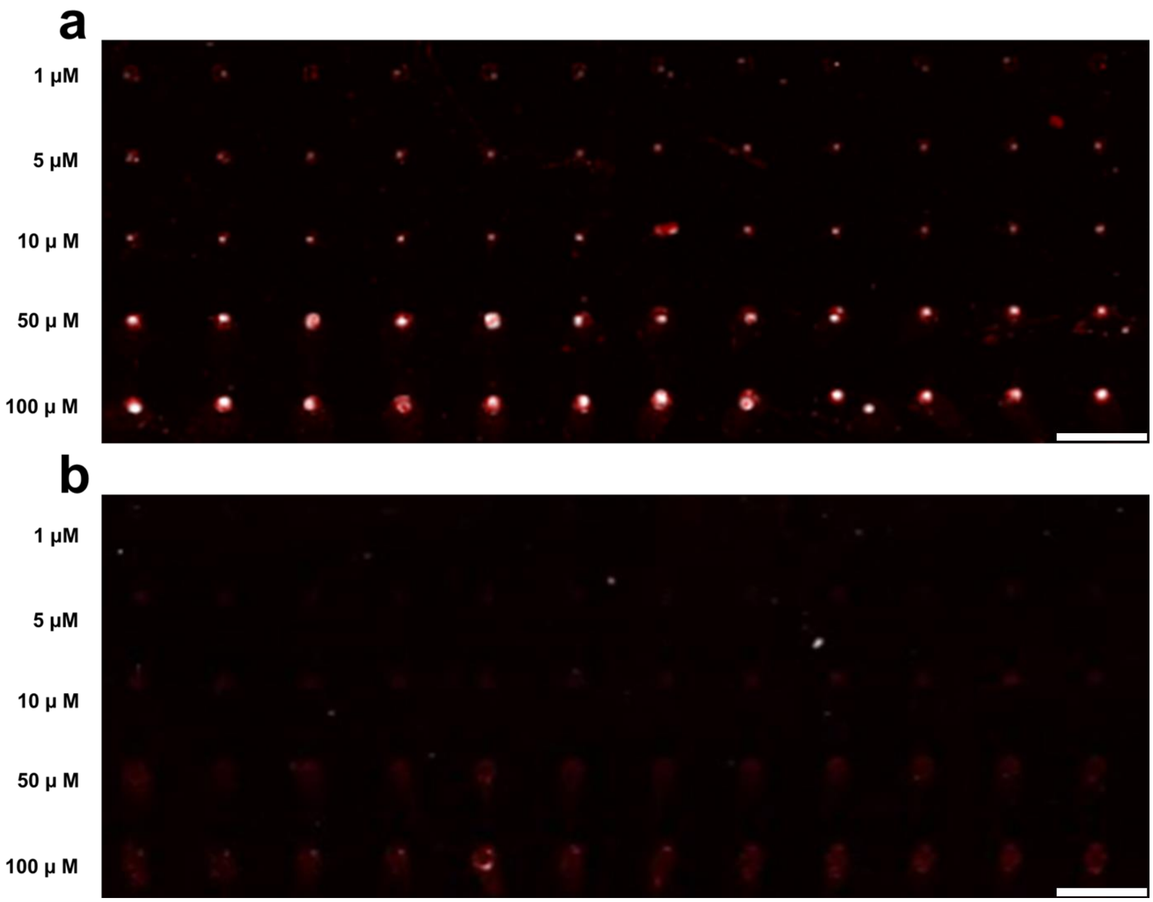

Supplementary Figure 10. Optimal immobilization concentration of i-motif DNA linkers for structure switchable DNA-based glycan chip. Scanned raw images for (a) hybridization Alexa Fluor® 647-conjugated oligonucleotides with surface-immobilized i-motif DNAs and (b) denaturation of Alexa Fluor® 647-conjugated oligonucleotides from surface-immobilized i-motif DNAs under acidic conditions (pH 4.5). Scale bar is 800 μm.

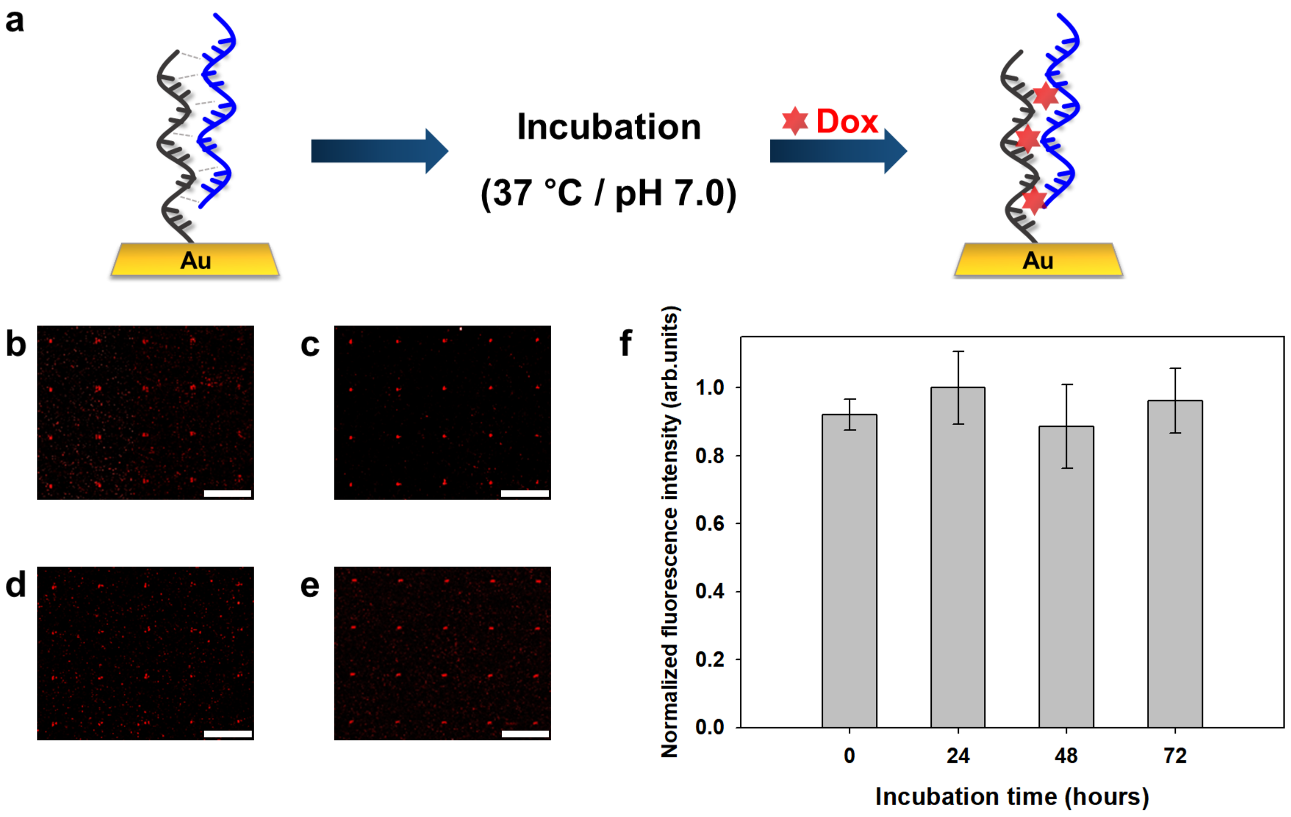

Supplementary Figure 11. Structural stability of the hybridized form of i-motif DNA under neutral conditions (pH 7.0). (a) Schematic illustration of an experimental protocol to test the structural stability of the hybridized form under neutral pH conditions. Scanned raw images for incubation times of (b) 0 h, (c) 24 h, (d) 48 h, and (e) 72 h (scale bar: 800 μm) and (f) their quantitative fluorescence intensity plot. Each value presents the mean ± SEM from twenty independent spots excluding the highest and lowest signals. Symbols: black helix, i-motif DNA; blue helix, complementary ssDNA; red star, doxorubicin. Source data are provided as a Source Data file.

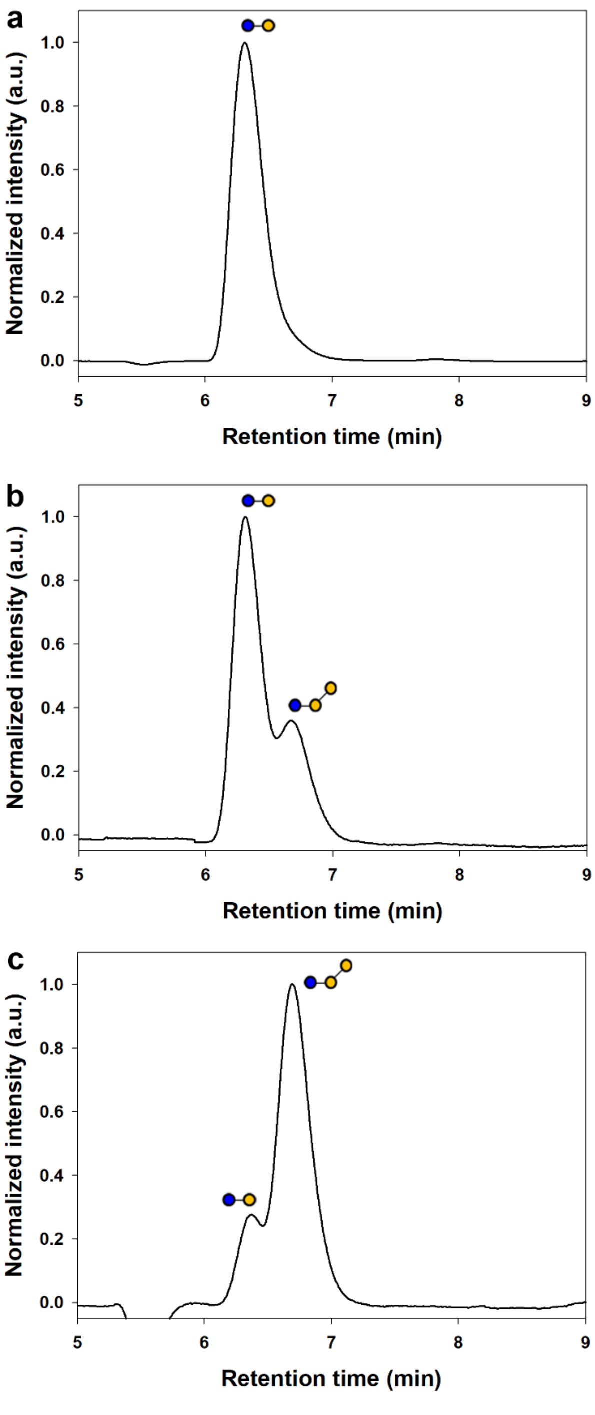

Supplementary Figure 12. Bio-LC analyses of on-chip biosynthesized Gb3 trisaccharide from lactose disaccharide by LgtC glycosyltransferase according to reaction time. Bio-LC chromatograms for reaction time of (a) 12 h, (b) 24 h, and (c) 48 h. Symbols: blue circle, Glc; yellow circle, Gal.[i] Source data are provided as a Source Data file.

---

[i] The products were analyzed by liquid chromatography (ICS-5000; Thermo Fisher Scientific) using a CarboPac PA100 column (4 mm × 250 mm; Dionex, Sunnyvale, CA, USA), isocratic elution mode with 100 mM sodium hydroxide, a flow rate of 0.25 ml/min, and an Ag/AgCl reference electrode for electrochemical detection.

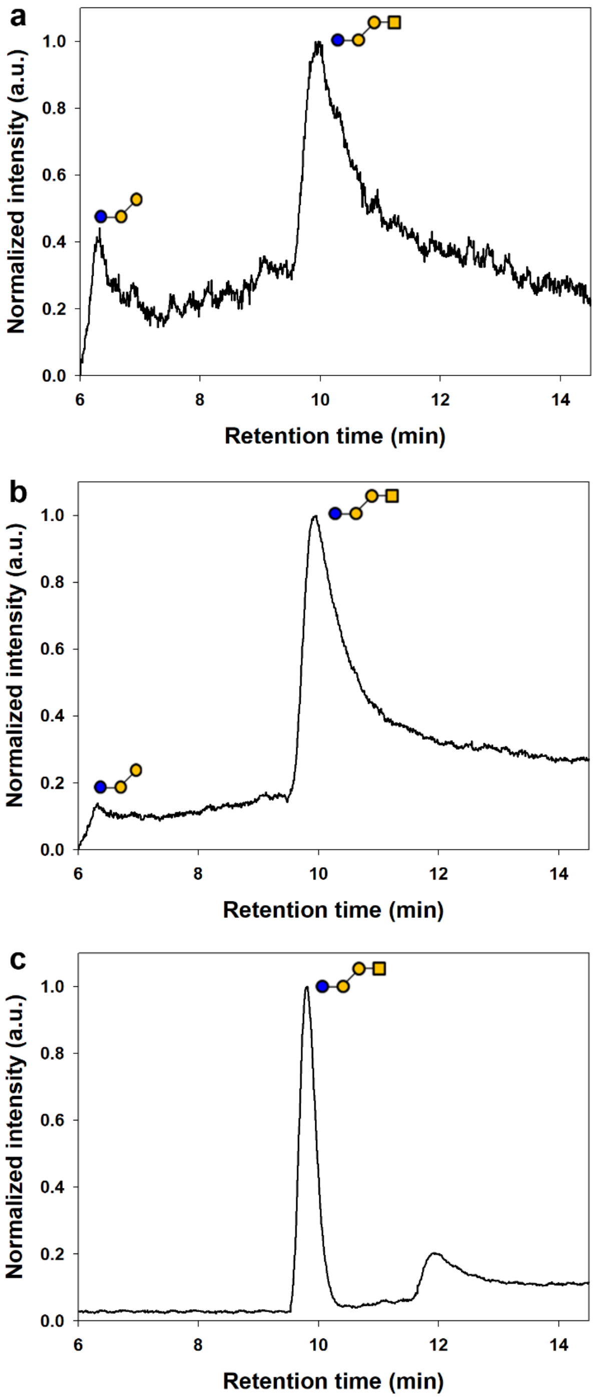

Supplementary Figure 13. Bio-LC analyses of on-surface biosynthesized Gb4 tetrasaccharide from Gb3 trisaccharide by LgtD glycosyltransferase according to reaction time. Bio-LC chromatograms for reaction time of (a) 12 h, (b) 24 h, and (c) 48 h. Symbols: blue circle, Glc; yellow circle, Gal; yellow square, GalNAc.[i] Source data are provided as a Source Data file.

---

[i] The products were analyzed by liquid chromatography (ICS-5000; Thermo Fisher Scientific) using a CarboPac PA100 column (4 mm × 250 mm; Dionex, Sunnyvale, CA, USA), isocratic elution mode with 100 mM sodium hydroxide, a flow rate of 0.25 ml/min, and an Ag/AgCl reference electrode for electrochemical detection.

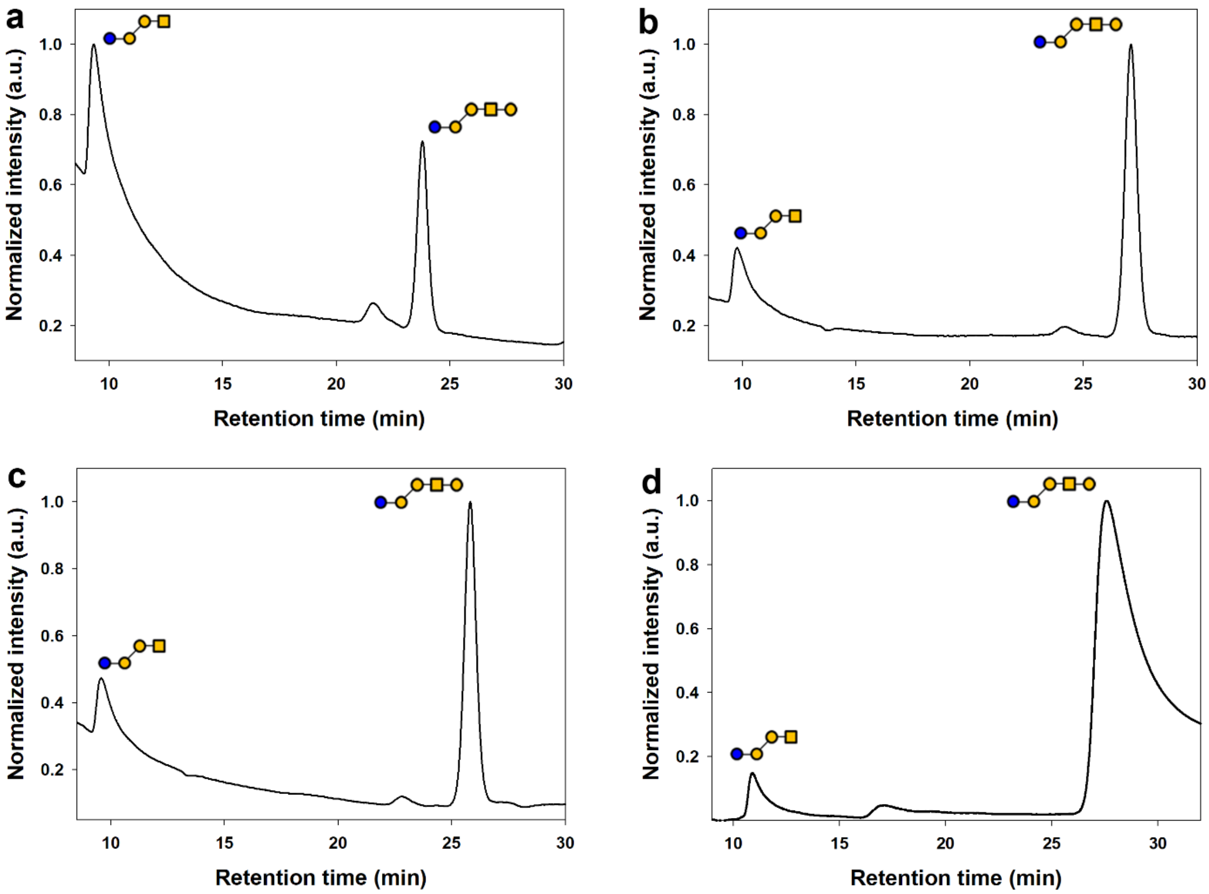

Supplementary Figure 14. Bio-LC analyses of on-surface biosynthesized Gb5 pentasaccharide from Gb4 tetrasaccharide by LgtD glycosyltransferase according to reaction time. Bio-LC chromatograms for reaction time of (a) 12 h, (b) 24 h, (c) 48 h, and (d) 72 h. Symbols: blue circle, Glc; yellow circle, Gal; yellow square, GalNAc.[i] Source data are provided as a Source Data file.

[i] The products were analyzed by liquid chromatography (ICS-5000; Thermo Fisher Scientific) using a CarboPac PA100 column (4 mm × 250 mm; Dionex, Sunnyvale, CA, USA), isocratic elution mode with 100 mM sodium hydroxide, a flow rate of 0.25 ml/min, and an Ag/AgCl reference electrode for electrochemical detection.

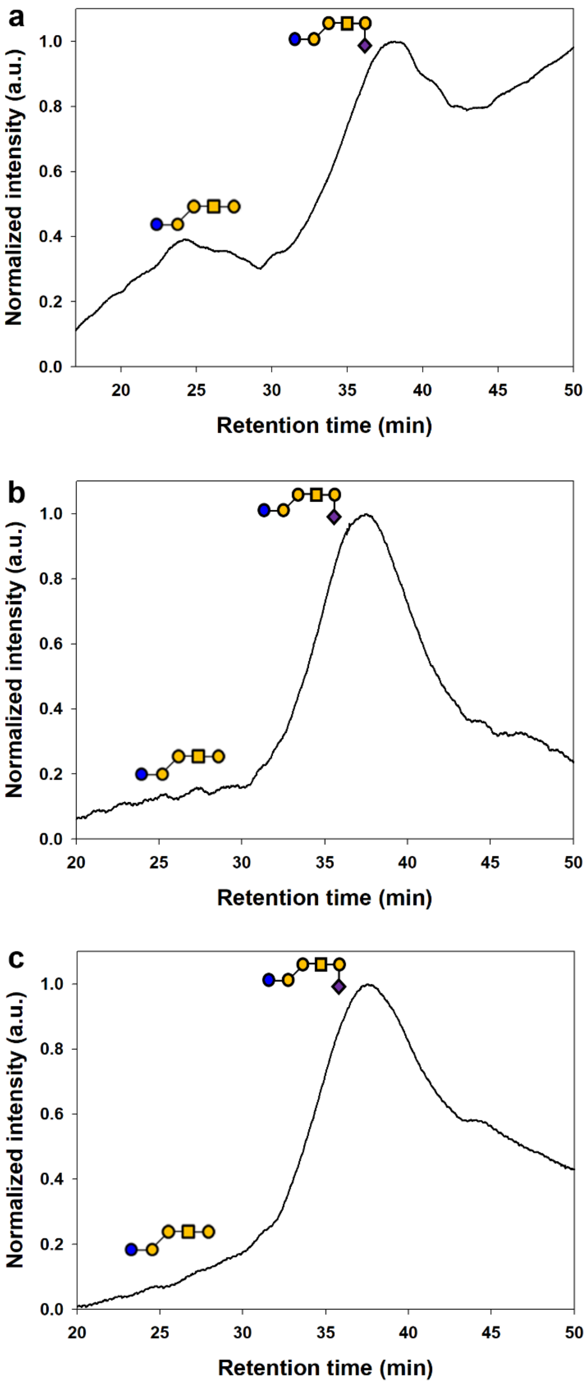

Supplementary Figure 15. Bio-LC analyses of on-surface biosynthesized SSEA-4 hexasaccharide from Gb5 pentasaccharide by α-2,3-SialT glycosyltransferase according to reaction time. Bio-LC chromatograms for reaction time of (a) 12 h, (b) 24 h, and (c) 48 h. Symbols: blue circle, Glc; yellow circle, Gal; yellow square, GalNAc; purple square, Neu5Ac.[i] Source data are provided as a Source Data file.

---

[i] The products were analyzed by liquid chromatography (ICS-5000; Thermo Fisher Scientific) using a CarboPac PA100 column (4 mm × 250 mm; Dionex, Sunnyvale, CA, USA), isocratic elution mode with 100 mM sodium hydroxide, a flow rate of 0.25 ml/min, and an Ag/AgCl reference electrode for electrochemical detection.

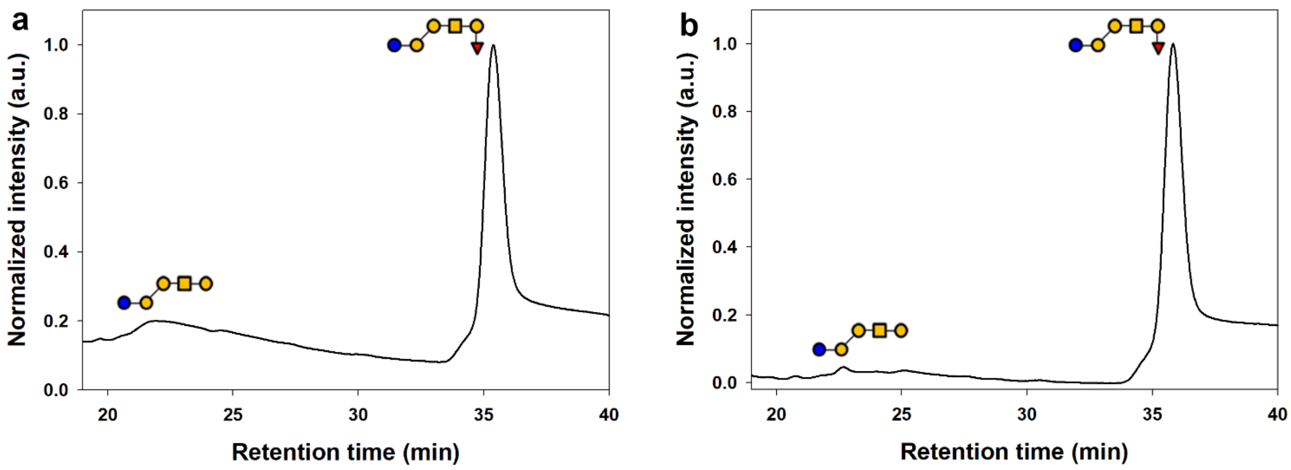

Supplementary Figure 16. Bio-LC analyses of on-surface biosynthesized Globo H hexasaccharide from Gb5 pentasaccharide by α-1,2-FucT glycosyltransferase according to reaction time. Bio-LC chromatograms for reaction time of (a) 24 h and (b) 48 h. Symbols: blue circle, Glc; yellow circle, Gal; yellow square, GalNAc; red triangle, Fuc.[i] Source data are provided as a Source Data file.

[i] The products were analyzed by liquid chromatography (ICS-5000; Thermo Fisher Scientific) using a CarboPac PA100 column (4 mm × 250 mm; Dionex, Sunnyvale, CA, USA), isocratic elution mode with 100 mM sodium hydroxide, a flow rate of 0.25 ml/min, and an Ag/AgCl reference electrode for electrochemical detection.

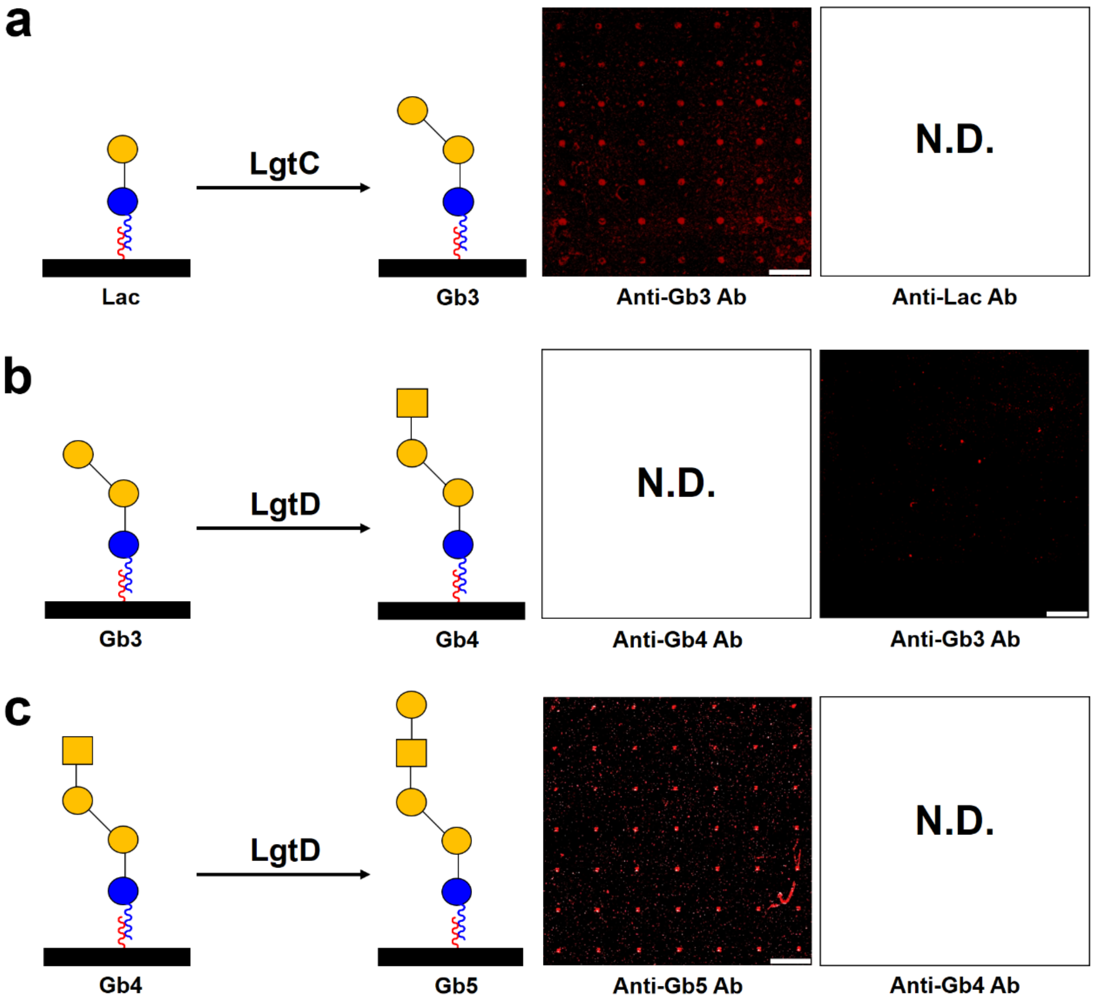

Supplementary Figure 17. Quality control for on-chip biosynthesized complex glycans using antibodies against starting materials and products. Schematic illustrations and scanned raw images for on-chip biosynthesized (a) Gb3 trisaccharide, (b) Gb4 tetrasaccharide, and (c) Gb5 pentasaccharide under optimized conditions (scale bar: 800 μm). Synthesized complex glycans were detected by using DyLight 650-conjugated monoclonal antibody or monoclonal antibody with Alexa Fluor® 647-conjugated polyclonal secondary antibody. Abbreviations: Anti-Gb3 Ab, Anti-Gb3 monoclonal antibody; Anti-Gb5 Ab, DyLight 650-conjugated anti-Gb5 monoclonal antibody; LgtC, α-1,4-galactosyltransferase; LgtD, β-1,3-N-acetylgalactosaminyltransferase/β-1,3-galactosyltransferase; N.D., not determined due to non-availability of commercial antibody. Symbols: blue circle, Glc; yellow circle, Gal; yellow square, GalNAc.

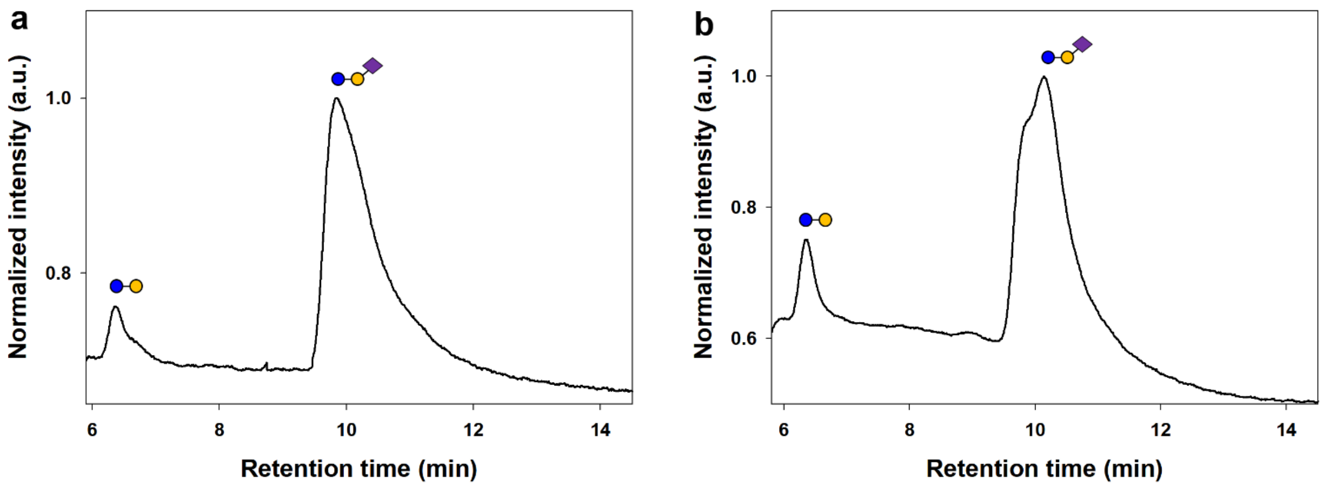

Supplementary Figure 18. Bio-LC analyses of on-surface biosynthesized GM3 trisaccharide from lactose disaccharide by α-2,3-SialT glycosyltransferase according to reaction time. Bio-LC chromatograms for reaction time of (a) 24 h and (b) 48 h. Symbols: blue circle, Glc; yellow circle, Gal; purple square, Neu5Ac.[i] Source data are provided as a Source Data file.

[i] The products were analyzed by liquid chromatography (ICS-5000; Thermo Fisher Scientific) using a CarboPac PA100 column (4 mm × 250 mm; Dionex, Sunnyvale, CA, USA), isocratic elution mode with 100 mM sodium hydroxide, a flow rate of 0.25 ml/min, and an Ag/AgCl reference electrode for electrochemical detection.

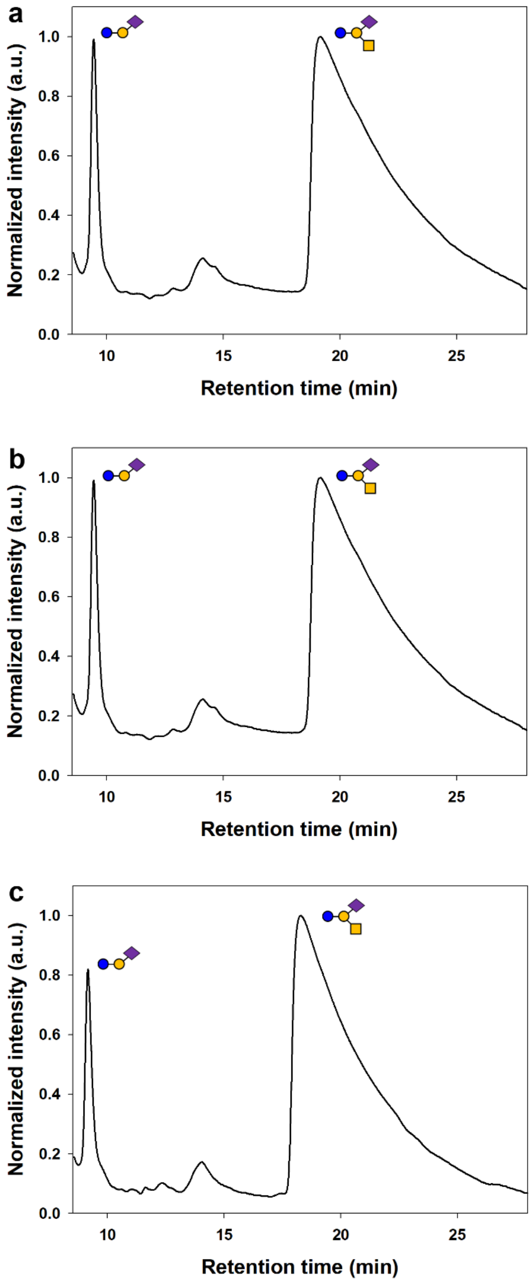

Supplementary Figure 19. Bio-LC analyses of on-surface biosynthesized GM2 tetrasaccharide from GM3 trisaccharide by *N*-acetylgalactosaminyltransferase (CgtA) according to reaction time. Bio-LC chromatograms for reaction time of (a) 72 h, (b) 120 h, and (c) 168 h. Symbols: blue circle, Glc; yellow circle, Gal; purple square, Neu5Ac; yellow square, GalNAc.[i] Source data are provided as a Source Data file.

[i] The products were analyzed by liquid chromatography (ICS-5000; Thermo Fisher Scientific) using a CarboPac PA100 column (4 mm × 250 mm; Dionex, Sunnyvale, CA, USA), isocratic elution mode with 100 mM sodium hydroxide, a flow rate of 0.25 ml/min, and an Ag/AgCl reference electrode for electrochemical detection.

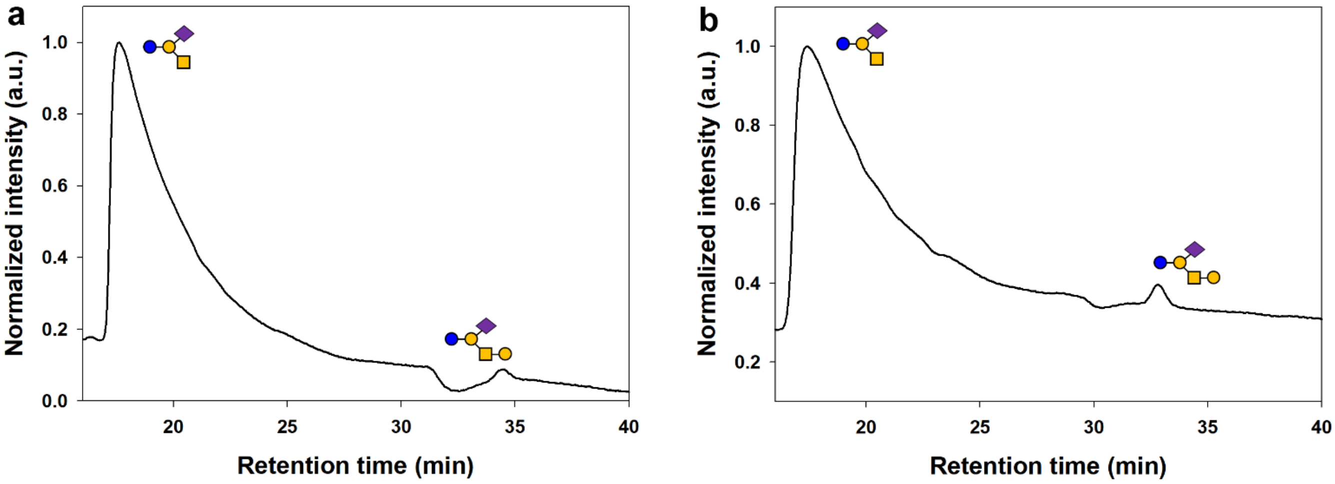

Supplementary Figure 20. Bio-LC analyses of on-surface biosynthesized GM2 tetrasaccharide from GM3 trisaccharide by galactosyltransferase (CgtB) according to reaction time. Bio-LC chromatograms for reaction time of (a) 72 h and (b) 120 h. Symbols: blue circle, Glc; yellow circle, Gal; purple square, Neu5Ac; yellow square, GalNAc.[i] Source data are provided as a Source Data file.

[i] The products were analyzed by liquid chromatography (ICS-5000; Thermo Fisher Scientific) using a CarboPac PA100 column (4 mm × 250 mm; Dionex, Sunnyvale, CA, USA), isocratic elution mode with 100 mM sodium hydroxide, a flow rate of 0.25 ml/min, and an Ag/AgCl reference electrode for electrochemical detection.

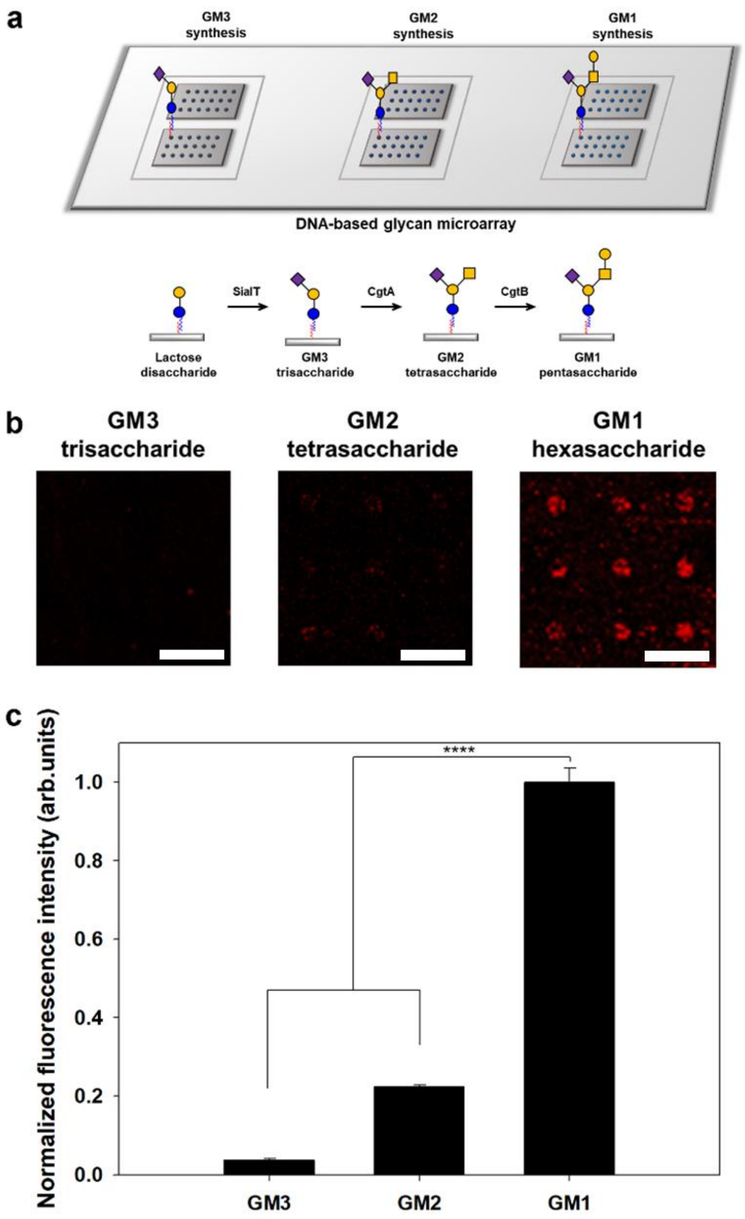

Supplementary Figure 21. Interaction analysis between on-chip biosynthesized complex glycans and cholera toxin B subunit. (a) Schematic presentation of the slide format for on-chip biosynthesis of GM3 trisaccharide, GM2 tetrasaccharide, and GM1 pentasaccharide on a lactose disaccharide-immobilized chip surface. (b) Scanned raw images (scale bar: 800 μm) and (c) fluorescence intensity plot for the interactions of cholera toxin B subunit with GM3 trisaccharide, GM2 tetrasaccharide, and GM1 pentasaccharide biosynthesized on the chip. Each value presents the mean ± SEM from twenty-five independent spots excluding the highest and lowest signals. Statistical significance was assessed using Student's unpaired *t*-test (****, $p < 0.0001$). Each value is the mean of twenty-five independent spots (excluding the highest and lowest signals), and the error bars represent the standard deviation. Symbols: blue circle, Glc; yellow circle, Gal; purple square, Neu5Ac; yellow square, GalNAc. Source data are provided as a Source Data file.

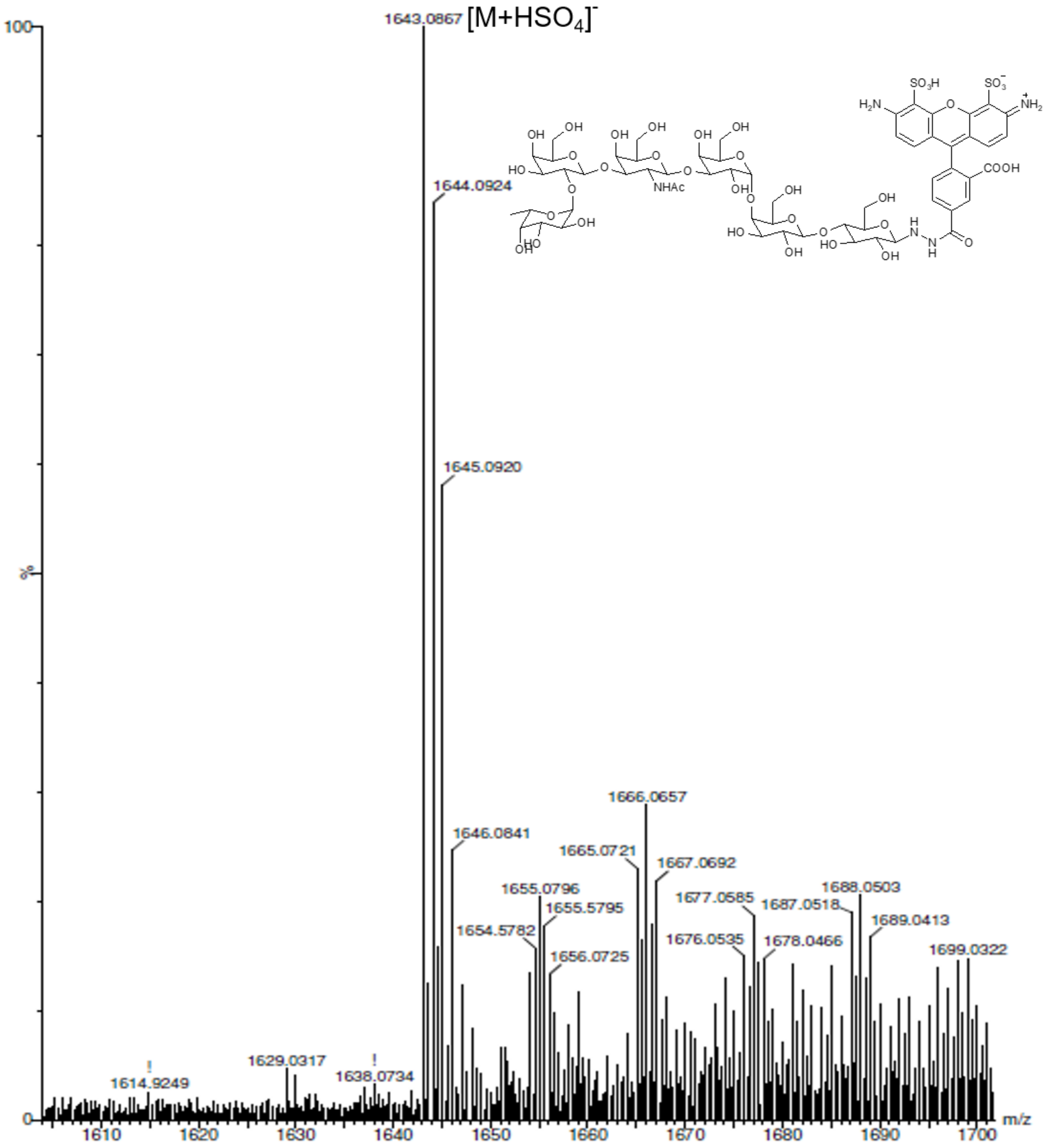

Supplementary Figure 22. LC-MS analysis of Globo H hexasaccharide-Alexa Fluor® 488 conjugate. LC-MS (m/z): $[M+HSO_4]^-$ calcd. for C59H79N5O39S2, 1642.38; found, 1643.0857.

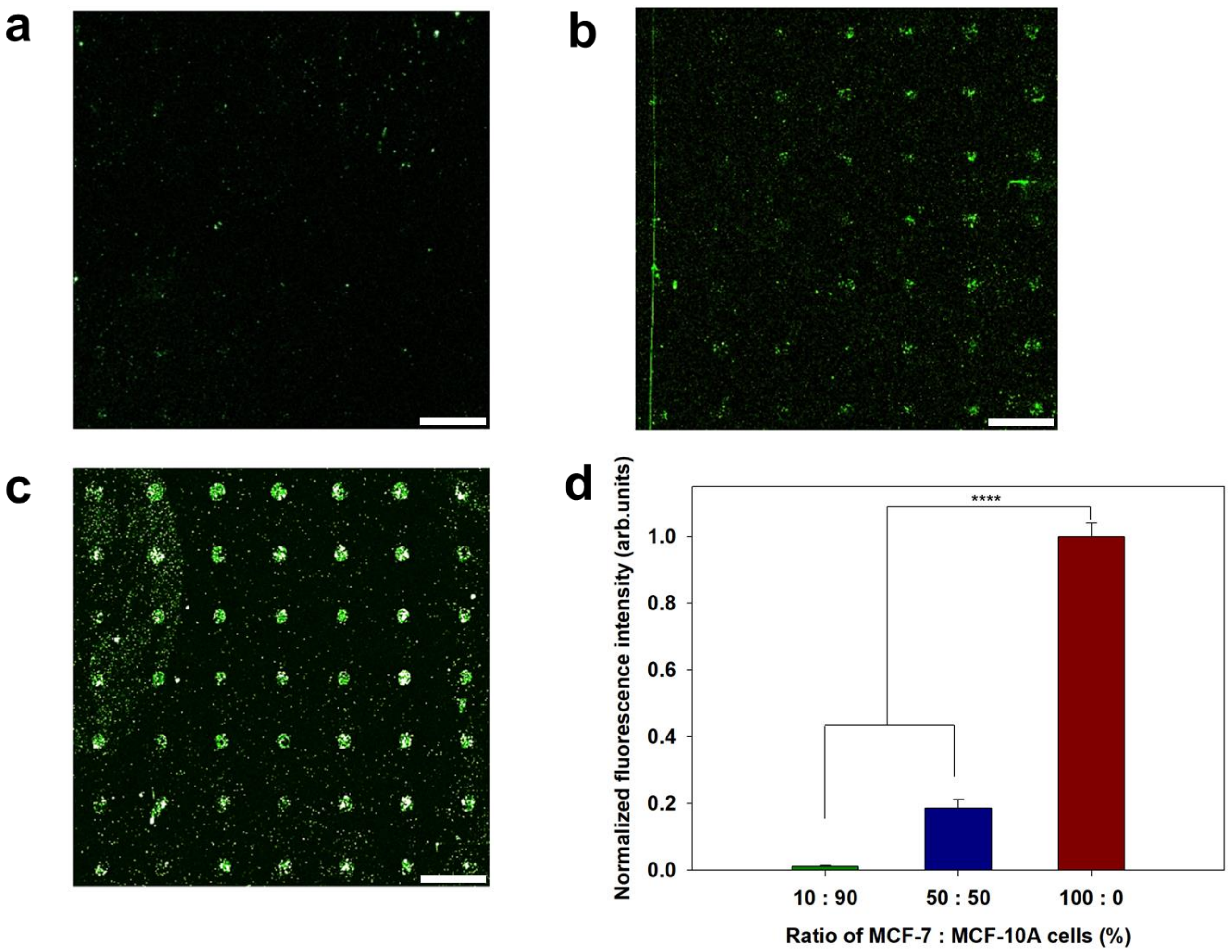

Supplementary Figure 23. Quantitative analysis for the binding of MCF-7 cancer cells to Globo H hexasaccharides on the glycan chip. (a-c) Scanned raw images (scale bar: 800 μm) and (d) fluorescence intensity plot for the direct binding of mixture of MCF-7 and MCF-10A cells with on-chip biosynthesized Globo H hexasaccharide. The number ratio (%) of MCF-7 cells to MCF-10A cells is (a) 10:90, (b) 50:50, and (c) 100:0. Each value presents the mean ± SEM from forty-nine independent spots excluding the highest and lowest signals. Statistical significance was assessed using Student's unpaired *t*-test (****, $p < 0.0001$). Source data are provided as a Source Data file.

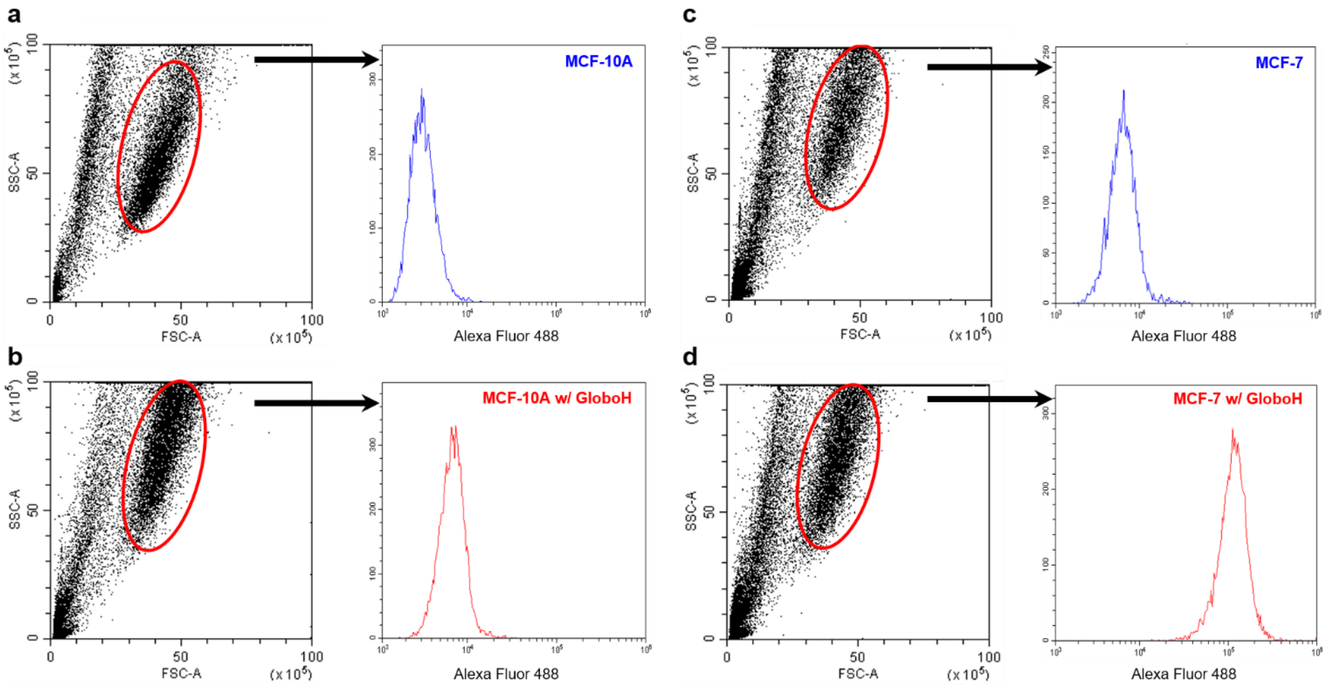

Supplementary Figure 24. Gating strategies used for cell sorting. Gating strategies to sort (a) sole MCF-10A normal cells, (b) MCF-10A normal cells with Globo H hexasaccharide, (c) sole MCF-7 cancer cells, and (d) MCF-7 cancer cells with Globo H hexasaccharide.
