# Supplementary material for: Glycan chip based on structure-switchable DNA linker for on-chip biosynthesis of cancer-associated complex glycans
Source: Nat Commun. 2021 Mar 2;12:1395. doi: 10.1038/s41467-021-21538-0 (PMC7925590; doi:10.1038/s41467-021-21538-0)
Supplement: Supplementary file 2 — Reporting Summary [file 41467_2021_21538_MOESM2_ESM.pdf]

nature research

Corresponding author(s): Hyung Joon Cha

Last updated by author(s): Jan 26, 2021

Reporting Summary

Nature Research wishes to improve the reproducibility of the work that we publish. This form provides structure for consistency and transparency in reporting. For further information on Nature Research policies, see our Editorial Policies and the Editorial Policy Checklist.

Statistics

For all statistical analyses, confirm that the following items are present in the figure legend, table legend, main text, or Methods section.

n/a	Confirmed	
☐	☒	The exact sample size (n) for each experimental group/condition, given as a discrete number and unit of measurement
☐	☒	A statement on whether measurements were taken from distinct samples or whether the same sample was measured repeatedly
☐	☒	The statistical test(s) used AND whether they are one- or two-sided<br>*Only common tests should be described solely by name; describe more complex techniques in the Methods section.*
☒	☐	A description of all covariates tested
☒	☐	A description of any assumptions or corrections, such as tests of normality and adjustment for multiple comparisons
☐	☒	A full description of the statistical parameters including central tendency (e.g. means) or other basic estimates (e.g. regression coefficient) AND variation (e.g. standard deviation) or associated estimates of uncertainty (e.g. confidence intervals)
☐	☒	For null hypothesis testing, the test statistic (e.g. F, t, r) with confidence intervals, effect sizes, degrees of freedom and P value noted<br>*Give P values as exact values whenever suitable.*
☒	☐	For Bayesian analysis, information on the choice of priors and Markov chain Monte Carlo settings
☒	☐	For hierarchical and complex designs, identification of the appropriate level for tests and full reporting of outcomes
☒	☐	Estimates of effect sizes (e.g. Cohen's d, Pearson's r), indicating how they were calculated

Our web collection on statistics for biologists contains articles on many of the points above.

Software and code

Policy information about availability of computer code

Data collection	No software was used to collect data in this study. After obtaining raw data through this study, it was analyzed as indicated below.
Data analysis	*Nuclear magnetic resonance (NMR) data: TopSpin 3.6.2 (Bruker, Karlsruhe, Germany)<br>*Array printing: AxSys (version 1. 79. 4. 0) (Cartesian Technologies, Irvine, CA)<br>*High pressure liquid chromatography (HPLC) system: TRILUTION® LC software (version 2 .1) (GILSON, Middleton, WI, USA)<br>*Laser scanner control and fluorescence image analysis: GenePix Pro 7 Software (Molecular devices, Sunnyvale, CA, USA)<br>*Quantitative analysis of synthesized product: Chromeleon 7 software (version 7. 2. SR4) (Thermo Scientific, Waltham, MA, USA)<br>*Graph creation: SigmaPlot 10.0<br>*Microsoft Excel (2016) was used for statistical analysis.<br>*Flow Cytometry: CyExpert (version 2. 3. 0)

For manuscripts utilizing custom algorithms or software that are central to the research but not yet described in published literature, software must be made available to editors and reviewers. We strongly encourage code deposition in a community repository (e.g. GitHub). See the Nature Research guidelines for submitting code & software for further information.

Data

Policy information about availability of data

All manuscripts must include a data availability statement. This statement should provide the following information, where applicable:

- Accession codes, unique identifiers, or web links for publicly available datasets
- A list of figures that have associated raw data
- A description of any restrictions on data availability

The authors declare that all data supporting the findings of this study are available within the article and its Supplementary Information files, or are available from

the authors upon request.
The following statement of Data Availability is mentioned in the manuscript after the methods section but before the references.
“DATA AVAILABILITY”
All data that support the findings of this study are available within the paper and supplementary information files. Source data are provided with this paper.

Field-specific reporting

Please select the one below that is the best fit for your research. If you are not sure, read the appropriate sections before making your selection.

- [x] Life sciences
- [ ] Behavioural & social sciences
- [ ] Ecological, evolutionary & environmental sciences

For a reference copy of the document with all sections, see nature.com/documents/nr-reporting-summary-flat.pdf

Life sciences study design

All studies must disclose on these points even when the disclosure is negative.

Sample size	The sample size was equal or larger than 3 in all cases. No method was used to calculate the sample size. We combined a number of analytical methods (e.g high pressure liquid chromatography, fluorescence image, etc) with standard statistical method. Based on the previous experiment results about on-chip enzymatic glycosylation (https://doi.org/10.1039/c8cc06526h), we have determined a sample size that can reduce variability and sufficiently confirm the consistency of the interaction analysis.
Data exclusions	The mean and standard error of the means in bar graphs were calculated excluding the highest and lowest signals to rule out the effect of signal intensity saturation.
Replication	We replicated our studies in several chip set-ups and on multiple occasions. A quantitative analysis of cancer-associated glycans synthesized on three independent chip surfaces was performed to verify data reliability. Experiments were performed with at least independent times yielding similar results and statistical significance was analyzed using Student's unpaired t-test.
Randomization	No randomization was used on the allocation of samples. To confirm whether enzymatic glycosylation is successfully performed on structure switchable DNA-based glycan chip, enzyme-treated and untreated chips were compared using lectins with known substrate specificity for the products. For quality control of on-chip glycan biosynthesis, antibodies with known binding specificity against starting glycans and products were used. Based on a previous study that there may be a major binding protein for Globo H hexasaccharide on the cancer cell membrane, glycan-binding specificity of MCF-7 cancer cells for on-chip biosynthesized Globo H series was assessed by using MCF-10A normal cell as control.
Blinding	The investigators were not blinded to the design of the study. Blinding was not possible as all glycans were biosynthesized in corresponding blocks divided on a chip surface and more than three independently prepared glycan chips were used to analyze glycan-binding of lectins, antibody, and cells. The interaction intensities are the mean of large numbers of spots on more than three independent glycan chips.

Reporting for specific materials, systems and methods

We require information from authors about some types of materials, experimental systems and methods used in many studies. Here, indicate whether each material, system or method listed is relevant to your study. If you are not sure if a list item applies to your research, read the appropriate section before selecting a response.

Materials & experimental systems

n/a	Involved in the study	
☐	☒	Antibodies
☐	☒	Eukaryotic cell lines
☒	☐	Palaeontology and archaeology
☒	☐	Animals and other organisms
☒	☐	Human research participants
☒	☐	Clinical data
☒	☐	Dual use research of concern

Methods

n/a	Involved in the study	
☒	☐	ChIP-seq
☐	☒	Flow cytometry
☒	☐	MRI-based neuroimaging

Antibodies

Antibodies used	All antibodies are commercially available. Anti-Globo H monoclonal antibody (VK9), DyLight 650-conjugated anti-Gb5 monoclonal antibody, Anti-Gb3 monoclonal antibody, and DyLight 650-conjugated anti-SSEA-4 monoclonal antibody were diluted in PBS buffer to a concentration of 25 ug/mL and used in antibody-binding analyses. Goat Anti-Mouse IgG H&L (Alexa Fluor® 647) was diluted in PBS buffer to a concentration of 50 ug/mL and used in antibody-binding analyses.<br>* Anti-Globo H monoclonal antibody (VK9): Catalog number 14-9700-82 (Invitrogen, Carlsbad, CA, USA)<br>* Goat Anti-Mouse IgG H&L (Alexa Fluor® 647): Catalog number ab150115 (Abcam, Cambridge, MA, USA)<br>*DyLight 650-conjugated anti-Gb5 monoclonal antibody (SSEA3 Monoclonal Antibody, DyLight 650): Catalog number MA1-020-D650

	(Invitrogen, Carlsbad, CA, USA)<br>*DyLight 650-conjugated anti-SSEA-4 monoclonal antibody(SSEA4 Monoclonal Antibody, DyLight 650): Catalog number MA1-021-D650(Invitrogen, Carlsbad, CA, USA)<br>*Anti-Gb3 monoclonal antibody: Catalog number A2506(Tokyo Chemical Industry Co., Ltd., Tokyo, Japan)
Validation	All antibodies were validated by the respective manufacturers. According to Invitrogen product datasheet, VK9 antibody is monoclonal antibody and recognizes Globo H hexasaccharide. VK9 has been reported for use in flow cytometric analysis, Western blotting, immunohistochemical staining of formalin-fixed paraffin embedded tissue sections, and microscopy. SSEA4 antibody is monoclonal antibody and conjugated to Dylight 650. DyLight 650-conjugated anti-SSEA-4 monoclonal antibody has been successfully used in ICC/IF and flow cytometry applications on human samples. SSEA3 antibody is monoclonal antibody and conjugated to Dylight 650. DyLight 650-conjugated anti-Gb5 monoclonal antibody has been successfully used in ICC/IF and flow cytometry applications on human samples. According to abcam datasheet, Goat anti-mouse IgG H&L (Alexa Fluor® 647) is goat polyclonal secondary antibody to mouse IgG-H&L and specific to mouse IgG. This labeled secondary antibody is used to detect mouse IgG, anti-Globo H monoclonal antibody (VK9) and anti-Gb3 monoclonal antibody. This product has been referenced in 60 publications. Abcam abpromise guarantee covers the use of goat anti-mouse IgG H&L (Alexa Fluor® 647) in the applications, including IHC-Fr, ICC/IF,ELISA,IHC-P, and flow cytometry.<br>According to TCI product datasheet, anti-Gb3 monoclonal antibody is purified mouse monoclonal antibody and recognizes glycolipid Gb3 trisaccharide. Gb3 monoclonal antibody has been reported for use in detection of Gb3 in blood cells and TLC-immunostaining.<br>Antibody details in online databases are as follows:<br>Anti-Globo H monoclonal antibody (VK9) : https://www.thermofisher.com/antibody/product/Globo-H-Antibody-clone-VK9-Monoclonal/14-9700-82<br>DyLight 650-conjugated anti-Gb5 monoclonal antibody : https://www.thermofisher.com/antibody/product/SSEA3-Antibody-clone-MC-631-Monoclonal/MA1-020-D650<br>Goat Anti-Mouse IgG H&L (Alexa Fluor® 647) : https://www.abcam.com/goat-mouse-igg-hl-alexa-fluor-647-ab150115.html<br>Anti-Gb3 monoclonal antibody : https://www.tcichemicals.com/KR/ko/p/A2506

Eukaryotic cell lines

Policy information about cell lines

Cell line source(s)	MCF7 (ATCC® HTB-22™), MCF 10A (ATCC® CRL-10317™)
Authentication	Cell line authentication was initially performed by ATCC. Further authentication was performed by microscopy, as all two cell lines used in this study (MCF7 and MCF 10A) have quite distinct morphology.
Mycoplasma contamination	The cell lines were not tested for mycoplasma contamination.
Commonly misidentified lines (See ICLAC register)	No commonly misidentified cell lines were used in this study.

Flow Cytometry

Plots

Confirm that:

- [x] The axis labels state the marker and fluorochrome used (e.g. CD4-FITC).
- [x] The axis scales are clearly visible. Include numbers along axes only for bottom left plot of group (a 'group' is an analysis of identical markers).
- [x] All plots are contour plots with outliers or pseudocolor plots.
- [x] A numerical value for number of cells or percentage (with statistics) is provided.

Methodology

Sample preparation	A detailed description of the sample preparation procedure is given in the Methods sections "Fluorescence-activated cell sorting (FACS) analysis"<br>Briefly, MCF-7 breast cancer cells were cultured in DMEM (high glucose) supplemented with 10% (v/v) heat-inactivated FBS, 100 U/mL penicillin, and 100 μg/mL streptomycin. MCF-10A normal breast cells were cultured with Mammary Epithelial Basal Medium, which contains bovine pituitary extract, hydrocortisone, human epidermal growth factor, insulin, gentamicin, and amphotericin-B. Both cell lines were incubated at 37 °C in a humidified atmosphere of 5% CO2 and 95% air, and they were subcultured every 3 days. After incubation, the cells were detached and centrifuged. After removing the supernatant, both cell lines resuspended in culture medium and were treated with synthesized Alexa Fluor® 488-conjugated Globo H hexasaccharide at 37 °C for 1 h in a humidified atmosphere of 5% CO2 and 95% air. Each solution was centrifuged to remove the remaining dye-conjugated Globo H hexasaccharide. After washing carefully with culture medium and DPBS, glycan-treated and nontreated cells resuspended in DPBS were placed into the wells of a noncoated 96-well plate. These cells were sorted by FACS (Beckman Coulter, Brea, CA, USA). Data were acquired and analyzed by using CytExpert software v 2.3.0 (Beckman Coulter).
Instrument	CytoFLEX Flow Cytometer (Beckman Coulter, Brea, CA, USA)
Software	CytExpert Software (Beckman Coulter, Brea, CA, USA)

Cell population abundance	No cell sorting was employed.
Gating strategy	1. FSC and SSC gating to exclude debris<br>2. Alexa fluor 488 gating to quantify Globo H-bound cells

☒ Tick this box to confirm that a figure exemplifying the gating strategy is provided in the Supplementary Information.
